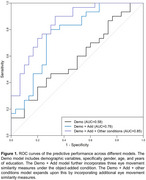# Global eye movement similarity as a neuropsychological marker of mild cognitive impairment in older adults in a visuospatial memory task

**DOI:** 10.1002/alz70857_098509

**Published:** 2025-12-24

**Authors:** Zhan Wang, Hong‐Zhou Xu, Jing Yu

**Affiliations:** ^1^ Southwest University, Chongqing, Chongqing, China

## Abstract

**Background:**

Previous studies have highlighted localized eye movement abnormalities in older adults with mild cognitive impairment (MCI) during visuospatial memory tasks. However, the role of global eye movement in the context of MCI remains untested. Using the framework of representational similarity, this study examines whether global eye movement patterns can reflect cognitive impairments in older adults, aiming to identify neuropsychological markers for MCI through the lens of global eye movement similarity.

**Method:**

The current study included two experiments. In Experiment 1, 36 cognitively normal older adults completed the adapted Visuospatial Memory Eye‐Tracking Task (VisMET), consisting of encoding and retrieval phases. During encoding, participants viewed a series of indoor scene images, each containing three objects. In the retrieval phase, the images were altered by either removing, adding, or repositioning an object within the scene. Participants were asked to indicate whether the image had changed and what type of change had occurred (removing, adding, repositioning). Similarity scores were calculated for each participant's fixation patterns and scanpaths between the encoding and retrieval phases for the same scenes. Generalized linear mixed models (GLMMs) were used to examine the relationship between these similarity scores and memory performance. In Experiment 2, 30 older adults with MCI and 30 healthy controls completed a 4‐min passive version of the task with no explicit memory task. A support vector machine (SVM) model with leave‐one‐out cross‐validation was then employed to assess whether the significant similarity indices from Experiment 1 could discriminate older adults with MCI from healthy controls.

**Result:**

In Experiment 1, similarity in eye movement patterns between encoding and retrieval significantly predicted memory performance, with the strongest effects in the adding condition. In Experiment 2, eye movement similarities combined with demographic variables successfully discriminated individuals with MCI from healthy controls, achieving an area under the curve (AUC) value of 0.85.

**Conclusion:**

Findings from Experiment 1 suggest that global eye movement similarity between encoding and retrieval effectively reflects older adults' memory performance, particularly in the adding condition. Experiment 2 further shows that these indices can distinguish older adults with MCI from health controls, highlighting the potential for MCI screening.